# Labour Market Regulation and Youth Unemployment in the EU-28

**DOI:** 10.1007/s40797-021-00154-3

**Published:** 2021-06-01

**Authors:** Giorgio Liotti

**Affiliations:** Department of Political Sciences, University of Naples Federico II, Via Rodinò 22, 80133 Naples, Italy

**Keywords:** Youth unemployment, Flexibility, European countries, Panel data model, J64, J40, C27

## Abstract

The rise of youth unemployment has been one of the most serious problems which policymakers have had to deal with over the last two decades. Neoclassical economic theory suggests that the deregulation (i.e. higher flexibility) of the labour market stimulates firms to hire young people and—therefore—reduces youth unemployment. The aim of this study is to empirically test the validity of this hypothesis, analysing data on youth unemployment and labour market regulation index (LMRI) for 28 European countries in the period between 2000 and 2018. The empirical results—using two different econometric techniques (time and fixed effects that allows to take into account the presence of heterogeneity of countries in the model and pooling mean group (PMG) estimator providing results about the short and long run relationship between LMRI and youth unemployment)—do not provide evidence in support of the neoclassical hypothesis. In particular, the effect of higher flexibility of the labour market is negative and statistically significant (at 1%) only when a dummy variable for the Eastern country group is included in the model. Vice-versa, the paper shows that higher economic growth and higher investment in active labour market policy represent the key variables to reduce the youth unemployment. In conclusion, the paper raises many doubts that the introduction of flexibility measures in itself can represent a useful tool to counteract the increase of youth unemployment in Europe.

## Introduction

The research concerning the reasons for the strong rise in (youth) unemployment—mainly in the Western European countries—has been at the centre of social, political and economic debate since the end of the 1980s (Lazear 1990; Layard et al. 1991; Pissarides and McCaster 1990). The hypothesis suggested by neoclassical labour market theory is that the higher unemployment was due mainly to the presence of more stringent rules of the labour market, which represented an obstacle to achieving full employment (Blanchard and Summers 1986; OECD 1994; Lazear 1990). It is worth pointing out that during the 1990s and the early years of the 2000s, the neoclassical hypothesis also found confirmation in several econometric papers (for more details see, among others, Nickell 1998; Nickell et al. 2005; Blanchard and Wolfer 2000; Blanchard et al. 2006; Hopenhayn and Rogerson 1993, Scarpetta 1996; Nickell and Layard 1999; Elmeskov et al. 1999; IMF 2003; Belot and Von Ours 2000).

The neoclassical theoretical arguments in favour of higher labour market flexibility are based on the fact that labour market flexibilization is a key factor to enhance productivity, increase a firm’s competition, favour economic growth and reduce unemployment (Jha and Golder 2008). In particular, it is possible to draw at least four reasons for why a high degree of rigidity in the labour market increases the unemployment level: (1) the existence of stringent labour market rules determines that, in equilibrium, workers’ wages are higher than their marginal product, leading, in this way, to a misallocation of resources; (2) higher labour market rigidity represents an obstacle to the adjustment of the labour market determined by the changes of the business cycle (Blanchard et al. 2006); (3) the rigidity of labour markets represents an economic “rent” from capital to labour that reduces the profitability of investors and discourages investment and economic growth (Calderon and Chong 2005); (4) finally, the rigidity of labour market institutions protects insider workers, preventing outsiders (especially young workers) from accessing the labour market (Lindebeck and Snower 1988).

According to these premises, labour market reforms have been introduced in many European countries since the later 1990s (Tridico 2018), with the aim of achieving three main objectives: (1) the introduction of “atypical” jobs (fixed and part-time contracts) to facilitate the entry of young people in the labour market; (2) lowering of the hiring and firing costs, allowing firms to increase their competitiveness on international markets and adjust the labour demand according to the business cycle (Zemanek 2010; Bernal-Verdugo et al. 2013; Lucifora et al. 2005; Ferreiro and Serrano 2013); (3) reducing employment security (Moreira et al. 2015), aiming to reduce the protection that insider workers enjoy, preventing the labour market segmentation described by insider–outsider theory^1^ (Lindbeck and Snower 1988; Blanchard and Summers 1986).

However, in this general context, it is important to point out that there is no consensus about the real effectiveness of labour market deregulation on employment outcomes (see among other Baker et al. 2004; Bassanini and Duval 2006; O’Higgins 2012, 1997; Dutt et al. 2015; Brancaccio et al. 2018; Arestis and González-Martínez 2015; Montenegro and Pages 2003; Ferreiro and Gomez 2019; Posner 2017).

In particular, there are at least three arguments in opposition to the neoclassical labour market theory hypothesis that deserve to be analysed. (1) According to Barbieri and Scherer (2009), higher deregulation does not contribute to a reduction in youth unemployment, but it determines only a substitution effect (that is, the substitution of typical employment with sub-protected workers (Cirillo et al. 2017; Ferreiro and Gomez 2018); (2) According to Kleinknecht (1998), policies aiming to provide greater flexibility in the labour market can have (maybe) some effect in the short run, but in the long run they are harmful for innovation, economic growth and employment; (3) According to Walwei (1996), labour market flexibility policies to reduce workers’ wages have detrimental effects for firms because they lead to “adverse” selection regarding workers (Akerlof 1984; Yellen 1984; Akerlof and Yellen 1986). Finally, it is also useful to recall that, according to the Keynesian school, the labour market has a passive role and that it depends on the level of aggregate demand. In this view, any changes in labour market institutions will not have an effect if they are not accompanied by expansionary economic policies (fiscal and monetary policies) that are implemented for the purpose of increasing the aggregate demand.

Considering the theoretical arguments pro and con, the motivation that underlies this paper is to try to evaluate whether the implementation of labour market flexibility has produced the desired effects on unemployment as stated by neoclassical theory or, vice versa, whether it is harmful for employment, as stated by heterodox scholars. This point is fundamental because, if the theoretical argument in favour of labour market flexibility is not able to achieve the objective for which it is implemented, then policymakers should find different solutions for the youth unemployment problem.

Compared to other papers in the same field, this paper: (1) does not focus on a measure capturing a specific aspect of the labour market (employment protection legislation, for example; see Liotti 2020 for more details), but on an indicator describing how and to what extent the labour market, as a whole, has been flexibilized; (2) tries to assess whether there are differences between short- and long-run effects of labour market flexibility on youth unemployment; and (3) focuses on the impact of single specific measures (i.e. sub-indicators) of flexibility on youth unemployment.

The paper is structured as follows. In Sect. 2, I discuss data analysis on youth unemployment and labour market regulation in 28 European countries. In Sect. 3, the empirical analysis is divided into three sub-sections: Sect. 3.1. The model and variables description, Sect. 3.2. Country fixed and Time fixed effects model and Sect. 3.3. PMG model. Section 4 is dedicated to a discussion on empirical results, and in Sect. 5 the general conclusions are drawn.

## Labour Market Regulation Index and Youth Unemployment in the EU-28: Data Analysis

In this section, the trend—over time—of two main variables (youth unemployment and labour market regulation) is analysed. The data focus on the following 28 European countries (Austria, Bulgaria, Belgium, Cyprus, Czech Republic, Croatia, Denmark, Estonia, Finland, France, Germany, Greece, Hungary, Ireland, Italy, Latvia, Lithuania, Luxembourg, Malta, the Netherlands, Poland, Portugal, Romania, Slovakia, Slovenia, Spain, Sweden and the UK) in the period between 2000 and 2018.

Regarding youth unemployment, according to the standard definition, the study takes into account the percentage of unemployed people aged 15–24. However, as showed by O’Higgins (1997), there is no single definition of youth unemployment, because it could vary depending on the aim of the research, and for this reason other age groups (for example 20–24 or 20–29) could also be considered.^2^

**Fig. 1:**
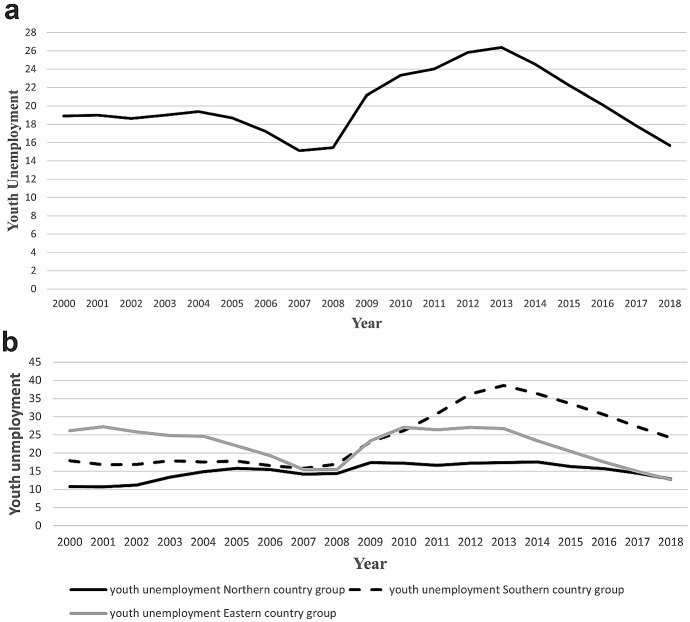
**a** Average youth unemployment rate in the EU-28. Soasdurce: Author’s elaboration on Eurostat data. **b** Average youth unemployment rate in Northern, Southern and Eastern country group in the period 2000–2018. Soasdurce: Author’s elaboration on Eurostat data

Figure 1a shows the trend of the (average) youth unemployment rate in EU-28 in the period 2000–2018.

The figure can be divided into three parts. In the first one, over the period 2000–2007, due to positive economic performance, youth unemployment reduced from 19 to 15.1%. In the second period, from 2008 to 2013, also as a consequence of the outbreak of economic crisis, youth unemployment rises by more than 11 percentage points, from 15.1 to 26.3%. Finally, in the third period, from 2014 to 2018, youth unemployment returned to the level of 2007 (about 15.7%).

However, because the data includes a set of heterogeneous countries, to identify the path of the unemployment rate it is necessary to divide the entities in the panel data into three different country groups: Northern Europe,^3^ Southern Europe^4^ and Eastern Europe.^5^ As we can see from Fig. 1b, these three groups exhibit a different path of the youth unemployment rate. Regarding the Northern country group, the figure shows that youth unemployment increased almost uninterruptedly until 2014, achieving a peak of 17.5% in that year. In contrast, since 2015 youth unemployment has slightly declined, and in the last year of observation—2018—it was about 14.5% (almost 4% points higher than 2000). Therefore, as shown in Fig. 1b, regarding the Northern country group, youth unemployment increased independently from the economic cycle, since it increased also before the 2008 crisis.

Considering the Southern European country group, it exhibits a stable youth unemployment rate until 2008 (about 17%), and then, as a consequence of the economic crisis, it increases to 36.3% in 2014. Therefore, in the last four years, it strongly declined, achieving 24.2% in 2018 (almost 7% points higher than the value in 2008).

Finally, analysing the Eastern European country group, the trend in youth unemployment shows a strong decline (from 26.1 to 15.4%) in the period between 2000 and 2008. Then, in 2009 and 2010, the rate increases to 27% and remains stable in 2013. After that, youth unemployment again falls to 12.7% in 2018 (about half of the level in 2013).

Now, after having analysed the trend in youth unemployment in the three country groups, I focus on the trend in labour market flexibility in the period 2000–2018. To capture the degree of flexibility of the labour market, I use the Labour Market Regulation Index (LMRI), elaborated by the Fraser Institute,^6^ which has two important advantages: (1) it allows us to take into account the changes in labour market institutions as a whole (Liotti 2020) and (2) it provides acceptable availability of data: its relevant data starting from 1970 is very suitable to analyse the long-run changes in labour market institutions that occurred over decades.

The LMRI index is an unweighted average of six measures—(1) hiring regulations and minimum wage; (2) hiring and firing regulations; (3) centralized collective bargaining; (4) hours regulations; (5) the mandated cost of worker dismissal; and (6) conscription—and its value varies from 1 to 10. The higher the value, the higher the degree of flexibility of the labour market.

**Fig. 2:**
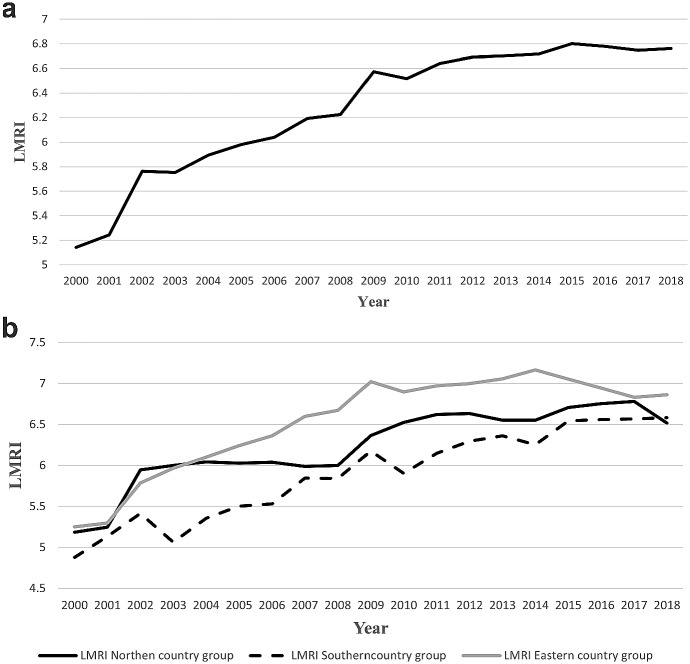
**a** Average labour market regulation index in the EU-28 in the period 2000–2018. Souasdrce: Author’s elaboration on Fraser Institute data. **b** Average labour market regulation in Northern, Southern and Eastern country groups in the period 2000–2018. Soasdurce: Author’s calculation on Fraser Institute data

Figure 2a shows the (average) value of the LMRI in the EU-28 from 2000 to 2018.

From Fig. 2a, it is possible to clearly individuate the change towards more flexibility of the labour market in the European continent. Indeed, the mean value of the LMRI increased from 5.1 in 2000 to 6.8 in 2018, with an increase equal to 1.7 (that is, 33.3%). However, Fig. 2a offers only a simple summary of the whole data set but says nothing about how much the three country groups have flexibilized their labour market institutions.^7^

Figure 2b shows the change in the LMRI in the three country groups in the period between 2000 and 2018.

Figure 2b shows four sets of interesting results: (1) the labour market has been flexibilized in all three country groups. (2) The Eastern country group shows higher flexibility of the labour market compared to the other two country groups. (3) Unlike the Southern and Eastern country groups, in which the flexibilization of the labour market never stopped, in the Northern country group, the LMRI remains stable in the period 2002–2008. This shows that, at least in this group, the change in the labour market accelerated mainly after the outbreak of the economic crisis. (4) Considering the difference between the last and the first year of observation, the change in the LMRI has been higher in the Southern country group (+ 1.70) compared to the Northern and Eastern country groups (respectively + 1.33 and + 1.60).

**Fig. 3:**
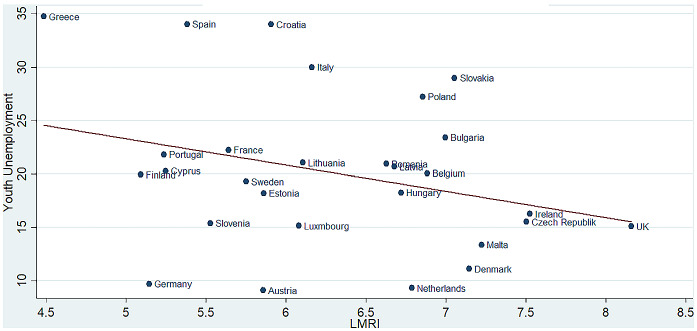
Correlation between youth unemployment and the LMRI in the EU-28. Soasdurce: Author’s calculation on Eurostat and Fraser Institute data

Finally, Fig. 3 plots the relation (for each country) between the average youth unemployment and the LMRI over the period 2000–2018.

As can be noted, the relation between the two variables is not very robust; indeed, even if for some countries (countries around the line) the correlation can seem strong, for others the correlation is less clear. Indeed, for example, Germany, Cyprus, Portugal and Spain have the same mean value of labour market regulation, but the youth unemployment rate is very different in these three countries. In particular, for the same LMRI, the youth unemployment rate in Spain and Portugal is respectively more than three and two times the youth unemployment rate in Germany. The same is true for Italy, Luxembourg and Lithuania: despite these countries having the same LMRI value, the youth unemployment rate in Italy is much higher than the last two countries.

Therefore, given that the effects of labour market flexibility on youth unemployment can be time varying and can vary across countries, this relationship deserves to be analysed empirically.

## Empirical Analysis

### The Model and Variables Description

To investigate the impact of the LMRI on youth unemployment in 28 European countries, the following equation is estimated:

1 $$ YU_{i,t} = \alpha_{i} + \beta_{0} Y_{i,t - 1} + \beta_{1} LMRI_{i,t - 1} + \mathop \sum \limits_{j = 0}^{1} \beta_{k} X_{i.t - j}^{\prime } + \delta_{n} Year + \varepsilon_{i,t} , $$

where $YU_{t}$ is the youth unemployment rate at time t, $Y_{i,t - 1}$ is the level of unemployment at time t-1, LMRI is the labour market regulation index, X' is the set of other variables affecting the youth unemployment rate, i are the entities, $\beta_{0.} \beta_{1.} \beta_{k}$ are the coefficients, $\varepsilon$ is the error term and $\alpha_{i}$ is the fixed effect considering the unobservable factors affecting the panel data entities. The empirical strategy assumes that the level of youth unemployment depends on its previous value, while the presence of the one-period lag of the main independent variable (LMRI) is coherent with the hypothesis in the literature that the causality goes from flexibility to unemployment and not vice versa. Besides the degree of flexibility of the labour market, other macroeconomic variables (that in the literature are assumed as determinants of youth and adult unemployment) are included in the model. These control variables are: (1) economic growth rate, (2) inflation, (3) government gross debt/GDP ratio, (4) current account balance, (5) active labour market policy per unemployed individual (ALMP/UNEMP) and (6) unemployment benefits. The source of the data is provided in the appendix (see Table 6 in “Appendix”).

The rationale for including these control variables is as follows (Table 1):

1. *Economic growth rate* According to Okun’s law, the increase in growth reduces the level of the unemployment rate. However, this view has been challenged in the past: indeed, Aghion and Howitt (1994) pointed out that the effect of economic growth on unemployment depends on two competing forces: the capitalization effect and the creative destruction effect. Regarding the first effect, economic growth raises the capitalization returns from creating jobs, and this leads to a reduction of the unemployment rate. The second effect, in contrast, reduces the duration of job matches, increases the job separation rate and discourages the creation of job vacancies. Therefore, the sign of the relationship between growth and unemployment depends on whether the first effect prevails on the second one or vice versa.
2. *Inflation* According to some authors (Choudry et al. 2012a; b), the inflation rate should contribute to reducing unemployment as, given that wages are predetermined through collective contracts, an increase in general prices reduces real wages and allows firms to increase labour demand and obtain a higher profit. However, I also need to consider the opposite view, which hypothesizes that inflation, especially if it is not kept under control by the authorities, could have a detrimental effect on unemployment. Indeed, a rise in the inflation rate, which reduces real wages, leads to reductions in domestic aggregate demand and in production levels. Without the intervention of external factors (for example, an increase in exports), inflation could contribute to the increase of youth unemployment.
3. *Government debt/GDP ratio* The hypothesis in the economic literature is that higher government debt/GDP has a negative effect on (youth) unemployment for two reasons: (1) the reduction of government debt/GDP forces countries to implement fiscal retrenchment measures, reducing in such a way the level of aggregate demand (Canale et al. 2014, 2019; Canale and Liotti 2015) and (2) higher government debt/GDP compromises the level of economic growth (Reinhart and Rogoff 2010). Therefore, according to this theory, the simultaneous estimate of these two variables can lead to biased results. However, looking at Table 1, we see that the correlation between growth and government debt/GDP is limited (− 0.23). This means that the effect of government debt/GDP on growth exists, but it is limited and certainly not able to bias the empirical results.
4. *Current account balance* This variable is one of the most important elements of aggregate demand. According to Keynesian theory, a positive current account balance means that the export level exceeds imports, and this should contribute to reducing youth unemployment. However, it could be pointed out that the current account balance is strictly linked to economic growth, and therefore in the empirical model the simultaneous estimate of these two variables can lead to biased results. This observation could be right if the correlation between these two variables were high, but according to Table 1, the correlation between growth and current account balance is very low (0.14). This means that the effect of current account balance on growth is limited and certainly not able to bias the empirical results.
5. *ALMP* Regarding the effect of ALMP, the literature seems to suggest that active labour market policies are negatively correlated with unemployment (Martin 2014). The theoretical assumption is that through schooling, training and experience, an unemployed person can increase the level of “human capital”, increasing in this way the probability to be more resilient in the face of different phases of the business cycle.
6. *Unemployment benefits* High unemployment benefits increase the unemployment level for two reasons: they reduce the job-searching intensity and they lower the economic cost of unemployment (Bassanini and Duval 2006).

**Table 1:** .

Variables	Youth Un	GDP gr	LMRI	Inflation	Debt/GDP	Current balance	ALMP	Unemployment benefits
Youth Un	1.00							
GDP gr	− 0.2294	1.00						
LMRI	− 0.1202	0.0722	1.00					
Inflation	− 0.0958	0.1366	− 0.0881	1.00				
Debt/GDP	0.4708	− 0.2313	− 0.1669	− 0.2471	1.00			
Current balance	− 0.1656	− 0.1742	0.1075	− 0.4641	− 0.0093	1.00		
ALMP	− 0.1908	− 0.1384	− 0.1916	− 0.2073	− 0.0456	0.4408	1.00	
Un. benefits	0.1009	− 0.3267	− 0.2894	− 0.2437	0.2432	0.3028	0.6051	1.00
VIF = 1.51								

Table 1 shows the matrix correlation among the variables included in the model. Many of the variables seem to be correlated with youth unemployment. In particular, the value is very high for government debt/GDP (0.4708). The correlation of the other variables is below 0.30. Finally, utilizing the VIF test (value equals 1.51) ensures that there is no multicollinearity among independent variables.

For estimating the impact, two panel data techniques are implemented: (a) first, I use a Country fixed and Time fixed effects panel data model. This empirical technique is important because it allows us to both take into account the presence of heterogeneity among entities and to control for unexpected variation or special events (for example an asymmetric shock) that may affect the dependent variable. (b) Then I use the pooling mean group (PMG) model elaborated by Pesaran and Smith (1995) to estimate the long-run effect of higher labour market flexibility on youth unemployment. This second methodology represents a robustness check of the results highlighted with the time and fixed effect approach.

### Country Fixed Effects and Time Fixed Effects Estimator

Here the effect of the LMRI on youth unemployment is estimated using the panel data model, taking into account both of the country fixed effects and time fixed effects. On the one side, country fixed effects must be used whenever the entities in the panel data show a certain degree of heterogeneity (for example, concerning the economic structures of each country) that could in some way bias the empirical estimates. The use of country fixed effects allows us to remove the effect of those time-invariant characteristics, in such a way that it is possible to assess the net effect of the independent variables on the dependent variable (Gujarai et al. 2017; Wooldrigde 2019). On the other side, time fixed effects must be used when we suspect that some unexpected events (for example asymmetric shocks) have affected and biased the empirical outcomes. Using time fixed effects allows us to depurate by unexpected events and to get unbiased results.

However, it is important to highlight that, given the strong heterogeneity of countries present in the data set, I also include two dummy variables: the first one helps to capture the differences about the effect of LMRI on youth unemployment in the Southern and Eastern country group compared to the Northern one (representing the reference group), while the second one allows us to investigate the effect of the LMRI on youth unemployment over the economic crisis period 2008–2014.

Table 2 shows the main empirical results.

**Table 2:** The effect of LMRI on youth unemployment in the EU-28

	(1)	(2)	(3)	(4)	(5)	(6)	(7)	(8)
$Youth \ Un._{t - 1}$	0.826*** (0.021)	0.830*** (0.020)	0.831*** (0.025)	0.842*** (0.021)	0.749*** (0.024)	0.825*** (0.021)	0.682*** (0.039)	0.789*** (0.027)
$Growth_{t - 1}$	− 0.410*** (0.049)	− 0.402*** (0.048)	− 0.417*** (0.050)	− 0.432*** (0.049)	− 0.325*** (0.051)	− 0.415*** (0.049)	− 0.507*** (0.063)	− 0.318*** (0.050)
$LMRI_{t - 1}$	− 0.178 (0.231)	− 0.234 (0.229)	− 0.186 (0.234)	− 0.122 (0.229)	− 0.156 (0.270)			− 0.074 (0.258)
$Inflation_{t - 1}$		0.153*** (0.044)						0.428*** (0.075)
$Debt/Gdp _{t - 1}$			− 0.004 (0.012)					− 0.021* (0.012)
$Current\_Bal_{t - 1}$				− 0.124*** (0.036)				− 0.030 (0.038)
$Unem\_Benefits_{t - 1}$					3.161*** (0.523)			3.264*** (0.489)
$ALMP_{t - 1}$					− 4.080*** (1.062)			− 3.440*** (1.060)
Dummy LMRI Northen countries						− 0.106 (0.234)		
Dummy LMRI Southern countries						− 0.063 (0.066)		
Dummy LMRI Eastern countries						− 0.117* (0.060)		
Dummy_LMRI_*t*-1__2008–2014							− 0.193 (0.517)	
Observations	497	497	487	497	445	497	224	441
R-Squared	0.9295	0.9318	0.9286	0.9340	0.9106	0.9299	0.8556	0.8881
No. of groups	28	28	28	28	27	28	28	27
Country fixed effects	Yes	Yes	Yes	Yes	Yes	Yes	Yes	Yes
Time fixed effects	Yes	Yes	Yes	Yes	Yes	Yes	No	Yes

Country fixed and Time fixed effects model. Dep. Var: Youth Unemployment

Cluster robust standard errors in parentheses

***, **, *Reject the null hypothesis at 1, 5 and 10 %

In Column 1 a basic model—including the one lag of economic growth beyond the LMRI and youth unemployment at time t−1—is estimated. It shows that the youth unemployment level depends on its past value (0.826),^8^ and this confirms that youth unemployment is a strongly persistent phenomenon, while the effect of economic growth and the LMRI is negative, respectively − 0.410 and − 0.178. However, it is important to note that the coefficient regarding the effect of the LMRI on youth unemployment is not statistically significant, while the coefficients of youth unemployment and economic growth are statistically significant at least at the 10% level, with R-squared equal to 93%.

Therefore, in Columns 2–5 I add other control variables to the model. In Column 2, inflation is added to the empirical regression: the results confirm the negative effect of economic growth (− 0. 402) and the LMRI (− 0.234) on youth unemployment, even though for the latter, I do not find statistical significance. On the other side, inflation contributes to the increase in youth unemployment (0.153). This result can be because higher inflation reduces the competitiveness of goods and services on international markets, reducing in such a way the aggregate demand and unemployment.

In Columns 3–5, government debt/GDP ratio, current account balance, ALMP and unemployment benefits are added to the model. Summarizing, results show that economic growth negatively affects youth unemployment in all estimations (the range is between − 0.325 (Column 5) and − 0.432 (Column 4), while the beneficial effect of the LMRI is in a range between − 0.122 (Column 4) and − 0.186 (Column 3), but it is never statistically significant.

Concerning other variables, unemployment benefits (Column 5) contribute to increase the youth unemployment (the value of coefficient is 3.161), therefore in line with the recent literature. A surplus in the current account balance (Column 4) and an increase in the government investments in ALMP (Column 5) reduce the unemployment level (− 0.124 and − 4.080, respectively) and, in this case, the hypothesis indicated in the literature is confirmed. At the same time, no statistically significant effect of the government debt/GDP ratio is found in the empirical results.

To complete the empirical analysis, in Column 6 I have added to the econometric model a dummy variable on the LMRI to analyse the different effect of the introduction of flexibility measures on three country groups, while in Column 7 I have included a dummy taking into account the effect of the LMRI on youth unemployment in the crisis period 2008–2014.

Column 6 of Table 2 shows interesting results: for the Northern country group, the effect is negative (− 0.106), but not statistically significant. Concerning the Southern country group, also in this case the effect is negative, but again it is not statistically significant. Only for the Eastern European country group does it seem that the flexibility measures have had a negative and statistically significant (at 1%) impact (− 0.117) in reducing the unemployment rate.

Column 7 shows a negative coefficient of the LMRI on youth unemployment in the crisis period; however, also in this case, the effect is not statistically significant.

Finally, in Column 8, I re-estimate the empirical model considering all independent variables simultaneously. The empirical results confirm the previous ones, with the only exceptions that now the negative coefficient regarding the effect of government debt/GDP is statistically significant (at 1 percent), while the coefficient regarding the current account balance is not statistically significant. However, the change in the significance of the coefficients could be due to the presence of some high variables (for example, there is a strict correlation between inflation and current account balance, − 0.4641).

According to Table 2, it seems that the LMRI in general does not affect youth unemployment, but it says nothing about the single sub-indicators that are at the base of these indices. So, I re-estimate Eq. 1 to assess whether some flexibility measures have had some beneficial effect on youth unemployment.

Table 3 shows the effect of each sub-indicator on youth unemployment.

**Table 3:** The effect of labour market regulation index sub indicators on youth unemployment

Variable	(1)	(2)	(3)	(4)	(5)
Youth unemployment_*t*−1_	0.822*** (0.022)	0.834*** (0.022)	0.826*** (0.027)	0.846*** (0.023)	0.738*** (0.025)
Growth_*t*−1_	− 0.431*** (0.052)	− 0.407*** (0.052)	− 0.432*** (0.052)	− 0.455*** (0.052)	− 0.346*** (0.052)
${\text{Inflation}}_{t - 1}$		0.171*** (0.048)			
${\text{Debt}}/{\text{Gdp }}_{t - 1}$			− 0.002 (0.013)		
${\text{Current}}\_{\text{Bal}}_{t - 1}$				− 0.136*** (0.039)	
${\text{Unem}}\_{\text{Benefits}}_{t - 1}$					3.304*** (0.528)
${\text{ALMP}}_{t - 1}$					− 4.380*** (1.102)
Hiring regulations and minimum wage_*t*−1_	− 0.090 (0.120)	− 0.153 (0.120)	− 0.110 (0.122)	− 0.067 (0.119)	− 0.066 (0.128)
Hiring and firing regulations_*t*−1_	− 0.082 (0.203)	− 0.188 (0.203)	− 0.074 (0.207)	− 0.125 (0.201)	− 0.120 (0.218)
Centralized collective bargaining_*t*−1_	0.163 (0.241)	0.017 (0.203)	0.132 (0.248)	0.122 (0.239)	0.108 (0.237)
Hours regulations_*t*−1_	− 0.154 (0.122)	− 0.144 (0.120)	− 0.151 (0.127)	− 0.110 (0.121)	− 0.311** (0.125)
Mandated cost of worker dismissal_*t*−1_	0.011 (0.142)	− 0.052 (0.141)	− 0.028 (0.145)	0.015 (0.142)	0.108 (0.139)
Conscription_*t*−1_	− 0.041 (0.071)	0.000 (0.071)	− 0.043 (0.072)	− 0.014 (0.070)	0.003 (0.072)
Observations	477	477	470	470	435
R-Squared	0.8593	0.8634	0.8639	0.8632	0.8785
No. of groups	28	28	28	28	28
Fixed effect	Yes	Yes	Yes	Yes	Yes
Time-effect	Yes	Yes	Yes	Yes	Yes

Country fixed effects and time fixed effects dep

Cluster robust standard errors in parenthesis

*Var* youth unemployment

***,**,*Reject the null-hypothesis at 1, 5 and 10%

Table 3 shows that some measures (hiring regulations, minimum wage, hiring and firing regulations, hours regulations and coscription) potentially could reduce youth unemployment, but none of the coefficients is statistically significant (with the only exception of hours regulation in column 5).

However, it is important to note that the empirical model using country fixed and time fixed effects can lead to biased results, because it does not take into account some characteristics of the panel data methodology (presence of the unit root and cointegration among variables). So, I next address these issues in order to check the robustness of these empirical results.

### PMG Estimator as Robustness Check of Results

To check the robustness of results derived through the country fixed and time fixed effects model, I estimate the relation between youth unemployment and labour market flexibility using the PMG methodology. The PMG estimator evaluates the long-run effect of the main independent variable on youth unemployment. Before implementing the empirical analysis, some econometric premises have to be considered: first, one of the most important issues, when large N and T are used, is that the idea of homogeneous slope parameters is inappropriate (Pesaran and Smith 1995; Blackburne and Frank 2007). For this reason, Pesaran and Smith (1995) proposed using a new empirical technique to estimate non-stationary dynamic panel data, in which it is supposed that the parameters are heterogeneous across the entities. This methodology is called the pooling mean-group estimator (PMG), and it consists of estimating N-time series regression and then taking the average values of coefficients (Pesaran and Smith 1995). Second, before estimating the general equation, I need to verify whether the variables of our primary interest are non-stationary at the level, are stationary at the differences and are cointegrated of rank 1. In the appendix (see Table 7 in “Appendix”), it is shown that the variables of the paper are all non-stationary at the level and stationary at the first difference. Moreover, we can see that all variables are cointegrated. These results are very important to avoid the possibility of estimating a spurious regression.

The equations to be estimated assume the long- and the short-run form. The long-run equation follows the ADRL $\left( {p; q_{1} \ldots q_{k} } \right)$ process using current and past values of the explanatory variables and is described by Eq. 3:

2 $$ YU_{i,t} = \alpha_{i} + \mathop \sum \limits_{j = 1}^{p} \beta_{i,j} YU_{i,t - j} + \gamma LMRI_{t - 1,i} \mathop \sum \limits_{j = 0}^{n} \delta_{i,j,} X_{i,t - j} + \varepsilon_{i,t} . $$

An important characteristic of this empirical approach is that, while in the short run it admits that the variables deviate from equilibrium, in the long run, all variables have to converge toward a stable equilibrium. Moreover, I consider the fixed effect option (DFE) to take into account the heterogeneity of the panel data.

Finally, it is possible to write out the error correction equation describing the short-run speed of adjustment in the following way:

3 $$ \Delta YU_{i,t} = \emptyset_{i} \left( {Y_{i,t - 1} - \theta_{i}^{\prime } X_{i,t} } \right) + \mathop \sum \limits_{j = 1}^{p - 1} \beta_{i,j} \Delta YU_{i,t - j} + \mathop \sum \limits_{j = 0}^{q - 1} \delta_{i,t} X_{i,t - j} + \varepsilon_{i,t} $$

where $\theta = - \left( {1 - \sum\nolimits_{j = 1}^{p} {\beta_{i,j} } } \right)$ is the error-correcting speed of adjustment term.

To guarantee that the variables converge in a long-run equilibrium, the parameter $\emptyset$ needs to assume negative values (for more detail see Blackburne and Frank 2007).

Table 4 shows the results of my estimations.

**Table 4:** The effect of LMR on youth unemployment in EU-28: dynamic panel data analysis with PMG estimator

Independent variables	(1)	(2)	(3)	(4)	(5)	(6)
*Long run*						
Growth_*t*−1_	− 2.114*** (0.262)	− 2.992*** (0.570)	− 1.403*** (0.498)	− 2.858*** (0.676)	− 1.339*** (0.314)	− 1.991*** (0.496)
$LMRI_{t - 1}$	− 2.051* (1.029)	− 0.686 (1.279)	− 1.097 (1.329)	− 0.826 (1.156)	− 1.948* (1.080)	1.387*** (1.466)
$Inflation_{t - 1}$		2.320*** (0.722)				3.139*** (0.762)
$Debt/Gdp _{t - 1}$			− 0.183 (0.117)			− 0.077 (0.125)
$Current\_Bal_{t - 1}$				− 1.331*** (0.504)		− 0.273 (0.325)
$Unem\_Benefits_{t - 1}$					10.614*** (2.499)	13.878*** (4.377)
$ALMP_{t - 1}$					− 19.140** (5.626)	− 15.095** (5.939)
*Short run*						
$\phi_{i}$	− 0.213*** (0.022)	− 0.183*** (0.021)	− 0.178*** (0.031)	− 0.175*** (0.029)	− 0.259*** (0.028)	− 0.198*** (0.027)
ΔGrowth_*t*−1_	− 0.048 (0.076)	0.011 (0.061	− 0.102 (0.074)	− 0.005 (0.062)	− 0.002 (0.026)	− 0.009 (0.053)
Δ$LMRI_{t - 1}$	0.030 (0.358)	0.142 (0.408)	− 0.160 (0.343)	− 0.323 (0.422)	− 0.069 (0.415)	− 0.448 (0.404)
Δ$Inflation_{t - 1}$		0.268** (0.112)				0.622*** (0.136)
Δ$Debt/Gdp _{t - 1}$			0.216*** (0.050)			− 0.015 (0.024)
Δ$Current\_Bal_{t - 1}$				− 0.223 (0.061)		− 0.054 (0.062)
$\Delta{Unem}\_Benefits_{t - 1}$					2.754*** (0.773)	2.751*** (0.856)
Δ$ALMP_{t - 1}$					− 4.967*** (1.402)	− 2.992** (1.272)
Groups	28	28	28	28	27	27
Observations	497	497	460	460	445	421

Dependent variable: Youth Unemployment

Cluster robust standard errors in parenthesis

***,**,*Reject the null-hypothesis at 1, 5 and 10 %

Looking to the long-run estimates, the results confirm that economic growth represents a key factor to reduce youth unemployment.^9^ The range of coefficients is in a range between − 1.339 (Column 5) and − 2.992 (Column 2), and they are always statistically significant.

Concerning the labour market flexibility, the results are controversial: the coefficients are negative and statistically significant at 1% for Column 1 (− 2.051) and Column 5 (− 1.948). However, if we look at Column 6, the sign of coefficient is reversed (1.387) and statistically significant; in other words, the higher LMRI leads to higher youth unemployment.

Concerning the other variables, on the one side, the detrimental effects of inflation (2.320), and unemployment benefits (10.614) are confirmed, while on the other side, a surplus in the current account balance (− 1.331) and investments in active labour market policies (− 19.140) regarding spending contribute to reducing youth unemployment. Regarding the coefficient of government debt/GDP, it is negative but not statistically significant. Furthermore, Column 6 reports the coefficients of the model that estimate all variables simultaneously: the only difference with respect to the general results is that the coefficient of current account balance is not statistically significant.

Finally, I re-estimate the effect of each sub-indicator of the LMRI in order to detect whether, in the long run, some flexibility policies have contributed to reducing youth unemployment.

Table 5 shows that, in the long run, some of the sub-indicators (hours regulation and Centralized collective bargaining) can have some limited effects, while regarding the other flexibility measures (Hiring regulations and minimum wage, Hiring and firing regulations, Mandated cost of worker dismissal, Conscription) are not statistically significant.

**Table 5:** The effect of labour market regulation index sub indicators on Youth Unemployment

Variables	(1)	(2)	(3)	(4)	(5)
*Long run*					
Growth_*t*−1_	− 2.189*** (0.381)	− 2.850*** (0.528)	− 1.500*** (0.464)	− 2.591*** (0.604)	− 1.357*** (0.342)
${\text{Inflation}}_{{{t} - 1}}$		2.470*** (0.716)			
${\text{Debt}}/{\text{Gdp}}_{{{t} - 1}}$			− 0.101 (0.094)		
${\text{Current}}\_{\text{Bal}}_{{{t} - 1}}$				− 1.932*** (0.679)	
${\text{Unem}}\_{\text{Benefits}}_{{{t} - 1}}$					9.787*** (2.234)
${\text{ALMP}}_{{{t} - 1}}$					− 17.403*** (5.090)
Hiring regulations and minimum wage_*t*−1_	0.108 (1.031)	− 0.091 (0.998)	− 0.140 (1.021)	− 0.024 (1.217)	0.497 (0.708)
Hiring and firing regulations_*t*−1_	0.896 (1.514)	0.262 (1.730)	0.939 (1.545)	0.313 (1.835)	0224 (1.430)
Centralized collective bargaining_*t*−1_	− 2.532 (2.003)	− 3.914* (2.307)	− 1.404 (1.820)	− 3.382 (2.810)	− 2.158* (1.206)
Hours regulations_*t*−1_	− 1.289 (0.915)	− 0.546 (0.990)	− 0.958 (0.911)	− 0.514 (1.129)	− 1.492*** (0.566)
Mandated cost of worker dismissal_*t*−1_	− 0.712 (0.756)	− 1.036 (0.847)	− 0.889 (0.983)	− 0.406 (0.954)	0.110 (0.627)
Conscription_*t*−1_	− 0.101 (0.312)	0.596 (0.470)	− 0.000 (0.373)	0.187 (0.514)	0.120 (0.259)
*Short run*					
$\emptyset$_*i*_	− 0.225*** (0.031)	− 0.184*** (0.028)	− 0.208*** (0.036)	− 0.167*** (0.036)	− 0.275*** (0.041)
ΔGrowth_*t*-1_	− 0.059 (0.076)	− 0.016 (0.060)	− 0.105 (0.076)	− 0.015 (0.043)	− 0.081 (0.063)
$\Delta Inflation_{t - 1}$		0.212** (0.093)			
$\Delta Debt/Gdp _{t - 1}$			0.206*** (0.049)		
$\Delta Current\_Bal_{t - 1}$				− 0.333 (0.065)	
$\Delta Unem\_Benefits_{t - 1}$					0.488 (0.878)
$\Delta ALMP_{t - 1}$					− 0.927 (2.051)
ΔHiring regulations and minimum wage_*t*−1_	0.205 (0.147)	0.212 (0.150)	0.231 (0.148)	0.201* (0.106)	0.142 (0.117)
ΔHiring and firing regulations_*t*−1_	0.283 (0.262)	0.474** (0.239)	0.279 (0.276)	0.235 (0.271)	0.542 (0.293)
ΔCentralized collective bargaining_*t*−1_	0.111 (0.334)	0.260 (0.268)	− 0.012 (0.337)	0.070 (0.323)	0.047 (0.312)
ΔHours regulations_*t*−1_	− 0.240 (0.153)	− 0.147 (0.155)	− 0.281 (0.175)	− 0.368** (0.158)	− 0.079 (0.109)
ΔMandated cost of worker dismissal_*t*−1_	− 0.444 (0.277)	− 0.112 (0.272)	− 0.402 (0.270)	− 0.519* (0.274)	− 0.498** (0.252)
ΔConscription_*t*−1_	0.004 (0.125)	− 0.107 (0.123)	− 0.064 (0.126)	− 0.146 (0.112)	− 0.039 (0.128)
Observations	477	477	477	477	477
No. of groups	28	28	28	28	28
Country Fixed effects	Yes	Yes	Yes	Yes	Yes

Cluster robust standard errors in parenthesis

PMG model Dep

*Var* Youth unemployment

***,**,*Reject the null-hypothesis at 1, 5 and 10 %

## Discussion on Empirical Results

This section is dedicated to a brief discussion comparing the empirical results obtained in this study with the relevant literature about labour market flexibility.

As discussed above, according to mainstream labour market theory, the main pillar of labour market institution reforms in the last 30 years was based on the postulate that high worker protection—preventing labour supply and labour demand from responding quickly to economic system changes—represents an obstacle to achieving full employment (Nickell 1998; Nickell et al. 2005; Blanchard and Wolfer 2000; Blanchard and Summers 1986; Blanchard et al. 2006). In other words, the existence of an inverse relation between the level of regulation and the unemployment rate represents the theoretical basis for justifying the necessity of deregulating the labour market through the adoption of flexibility measures (Lee 2000; European Commission 2009; Bernal-Verdugo et al. 2013; Bruno et al. 2017).

The results of this study point out that the hypothesis according to which higher labour market flexibility leads automatically to a decline in youth unemployment lacks empirical support. There could be several reasons, and I will deal with two in particular: (1) a possible explanation for this result could be due to the presence of high turnover among young workers. In other words, according to Barbieri and Scherer (2009), the result could be determined from a kind of substitution effect, in which firms continuously hire and fire using tax relief or other government incentives to reduce costs and make their goods and services more competitive in the international market. In this view, there is no stimulus for firms to increase the skills and human capital of young workers, who remain trapped in a continuous and growing precariousness trap (O’Higgins and Moscariello 2017). (2) Another reason that could justify the lack of empirical support for the neoclassical hypothesis is based on the deficit of aggregate demand. This argument derives from Keynesian effective demand theory and can be explained as follows: If one of the main objectives of higher labour market flexibility is to ease the access of young workers and reduce the hiring costs for firms, this policy, given the lack of stimulus of aggregate demand, becomes ineffective. Firms produce only if they have some expectation of selling what they produce, and the hiring of workers is subject to these conditions; but if there is an aggregate demand gap that makes firms have negative expectations about the future of the economy, the adoption of automatic labour market flexibility measures will fail to achieve the objectives for which they have been implemented. In this context, expansionary fiscal and/or monetary policies mixed with some flexibility measures could provide some beneficial effects not only for young employment but for employment in general.

In conclusion, looking at the general results, it must be noted that labour market flexibility cannot be considered a “panacea” for resolving the unemployment problem. The implementation of flexibility measures should be targeted, taking into consideration the whole economic, social and political structure of each individual country.

## Concluding Remarks

This study analysed the effect of higher labour market flexibility on youth unemployment, taking into account data on 28 European countries in the 2000–2018 period. The economic literature has suggested that higher labour market flexibility represents one of the main pillars to reduce (youth) unemployment. Indeed, a rigid labour market prevents the adjustment of the labour supply and labour demand according to the specific phase of the business cycle, increases production costs for firms and reduces the competitiveness of goods and services at the international level, and this leads to a high level of unemployment.

The European Union has adopted reforms that have reduced labour market rigidity; these reforms have reduced the so-called “security in employment” and have concerned several areas such as hiring and firing regulations, dismissal costs and minimum wages. The degree of flexibility is captured by the so-called labour market regulation index (LMRI) that considers the changes of labour institutions due to the reforms adopted by governments over time.

The aim of the study has been to empirically test the validity of the hypothesis of a beneficial effect of higher labour market flexibility on youth unemployment. From the empirical results, using two different empirical techniques (time and fixed effect model and PMG estimator), arise many doubts. Indeed, the empirical analysis shows that there is little or no support in favour of the neoclassical hypothesis stating that higher labour market flexibility is able to reduce youth unemployment.

Considering the other variables included in my empirical model, I find that growth, active labour market policies and a positive current account balance contribute to reducing youth unemployment, while inflation and unemployment benefits have detrimental effects on our main dependent variable.

In this regard, it is worth spending some words on the effect of growth on youth unemployment. That economic growth allows for the reduction of (youth) unemployment is not news, because this result—over time—has been confirmed empirically in several previous studies (Choudry et al. 2012a; b; Bruno et al. 2017); rather, much discussion revolves around the debate of how to create an environment favourable to economic growth. Here I will briefly focus on one of the most important factors that can create a favourable environment for economic growth, that is, fiscal policy. In this sense, according to Schubert and Turnovsky (2017), fiscal expansion stemming from an increase in government investment can achieve higher long-run economic growth and a lower long-run unemployment rate.

This policy could be particularly useful for the European Union, whose economy is being held back by the COVID-19 pandemic. In this context, the recovery funds that the European Commission will make available to countries that have particularly downsized their economies (for example Italy and Spain) should be used for government investments in strategic sectors (green economy, digitalization, R&D, innovation of industrial production and education), accompanied by higher spending for ALMP that allows young people easier access to the labour market, without reducing the social rights of workers.

In conclusion, policymakers should target public policies to guarantee fiscal incentives to stimulate firms to hire young people with a permanent contract, preventing these workers from falling into the trap of precariousness.

## Appendix

See Tables 6 and 7.

**Table 6:** Variables description and data source

Dependent variable	Definition	Source
Youth Unemployment	Youth (15–24 years) unemployed labour force/youth labour force	EUROSTAT
Independent variables		
GDP growth rate	Annual percentages of constant price GDP are year-on-year changes	IMF
Labour Market Regulations Index	The LMR index is an un-weighted average of these six measures and its value varies from 1 to 10	Fraser Institute
Inflation	Annual percentages of average consumer prices are year-on-year changes	IMF
Government debt/GDP	Gross debt consists of all liabilities that require payment or payments of interest and/or principal by the debtor to the creditor at a date or dates in the future	IMF
		*Data for Greece, Luxembourg and Slovakia Republic available on Eurostat website
Current account balance	Current account is all transactions other than those in financial and capital items	IMF
ALMP/UNEMP	Expenditure on active labour market policies per unemployed individual	OECD
		*Data for Bulgaria, Croatia, Malta and Romania available on Eurostat website
Unemployment benefits	Out-of-work income maintenance and support	OECD
		*Data for Bulgaria, Croatia, Malta and Romania available on Eurostat website

**Table 7:** Unit root test and cointegration test

Test						
Individual effects, and individual trend						
Variables	HAD	LLC	IPS	ADF	PP	Breitung
Youth Un	4.773***	− 1.968**	0.004	58.027	21.900	− 0.791
LMR	9.994***	− 0.146	− 0.880	61.822	85.636***	− 0.508
GDP	6.752***	− 1.692**	0.439	36.695	145.076***	− 2.997***
Inflation	9.126***	− 1.146	− 2.330***	66.064	126.225***	− 2.716***
Government debt/GDP	6.206***	− 2.815***	0.450	48.149	21.332	1.363
Current account balance	7.051***	− 2.379***	− 0.587	57.349	52.906	− 2.080**
Unemployment benefits	7.148***	− 3.295***	− 0.713	59.889	26.829	− 2.623***
Almp	7.848***	0.515	− 0.514	51.559	51.9681	0.058

First differences						
Individual effects, and individual trend						
Youth Un	5.892***	− 3.868***	− 3.025***	91.2649***	140.225***	− 5.539***
LMR	5.358***	− 0.441	− 4.096***	90.073***	317.986	− 3.851***
GDP	24.742***	− 2.515***	− 5.190***	104.353***	511.712***	− 9.627***
Inflation	14.091***	− 1.462*	− 4.903***	101.751***	437.558***	− 2.895***
Government debt/GDP	7.955	− 3.887***	− 2.240**	81.508**	85.901***	− 3.338***
Current account balance	7.371***	− 6.863***	− 6.284***	135.479***	308.084***	− 7.194***
Unemployment benefits	6.349***	− 9.789***	− 5.006***	117.390***	173.635***	− 8.899***
Almp	8.906***	− 2.823***	− 2.120**	71.942*	189.195***	− 1.857**
Cointegration test						
Kao Residual test for cointegration						
Ho: No cointegration, Ha: Some panels are cointegrated						

	Statistics	p value
ADF	− 4.444***	0.000

The tests are: Hadri, 2000 (HAD); Levin, Lin and Chu, 2002 (LLC); Im, Pesaran and Shin, 2003 (IPS); ADF Fisher χ^2^ (ADF); PP Fisher χ^2^ (PP) due to Maddala and Wu, 1999 and Breitung, 1999). In Hadri the null is that the variable is stationary. ***, **, and * reject the null at 1%, 5% and 10% respectively

*Youth unemployment* The no-stationary at level is confirmed by five tests (IPS, ADF, Breitung, PP and Hadri). The stationary at the differences is confirmed by the following five tests (LLC, IPS, ADF, PP and Breitung), *LMRI* the no-stationary at level is confirmed by five tests (LLC, IPS, ADF, HAD and Breitung. The stationary at the differences is confirmed by the following three tests (IPS, ADF and Breitung), *Growth rate* The no-stationary at level is confirmed by three tests (IPS, ADF and Hadri). The stationary at the differences is confirmed by the following five tests (LLC, IPS, ADF, PP and Breitung), *Inflation* the no-stationary at level is confirmed by three tests (LLC, ADF and Hadri). The stationary at the differences is confirmed by the following five tests (LLC, IPS, ADF, PP and Breitung), *Government debt/GDP* The no-stationary at level is confirmed by five tests (IPS, ADF, Breitung, PP and Hadri). The stationary at the differences is confirmed by the following five tests (LLC, IPS, ADF, PP and Breitung), *Current account balance* The no-stationary at level is confirmed by four tests (IPS, PP, ADF and Hadri). The stationary at the differences is confirmed by the following five tests (LLC, IPS, ADF, PP and Breitung). *Unemployment benefits* The no-stationary at level is confirmed by four tests (IPS, PP, ADF and Hadri). The stationary at the differences is confirmed by the following five tests (LLC, IPS, ADF, PP and Breitung), *ALMP* The no-stationary at level is confirmed by all six tests (LLC, IPS, Breitung, PP, ADF and Hadri). The stationary at the differences is confirmed by the following four tests (LLC, IPS, PP and Breitung). Kao test for cointegration rejects the hypothesis of no cointegration among variables used in the empirical analysis

## Footnotes

1 According to this theory, insider workers enjoy an “invisible” power that they could exert on the firm in such a way as to hold some benefits and create a barrier to the entry of young people in the labour market.

2 However, it is worth pointing out that youth unemployment represents only one of several indicators capturing the “dramatic” situation of young people in the modern era. Indeed, other indicators that would deserve to be analysed are: (a) youth employment (defined as the number of young employed persons as a percentage of the youth population; for more details see ILO 2009), (b) NEET (the sum of young persons who are not in employment, education or training; for more details see Caroleo et al. 2020) and (c) discouraged workers among youth (defined as the sum of all persons who are without work and available for work but did not seek work; see Dagsvik et al. 2013).

3 Austria, Belgium, Denmark, Finland, France, Germany, Luxembourg, the Netherlands, Sweden and the UK.

4 Cyprus, Greece, Ireland, Italy, Malta, Portugal and Spain.

5 Bulgaria, Croatia, Czech Republic, Estonia, Hungary, Latvia, Lithuania, Poland, Romania, Slovakia and Slovenia.

6 https://www.fraserinstitute.org/economic-freedom/dataset?geozone=world&page=dataset.

7 For details about the flexibility measures of the labour market in Europe, see, among others, Moreira et al. (2015), Cirillo et al. (2017) and Eichhorst et al. (2017).

8 In all estimations, the cluster robust standard errors are used.

9 Since the PMG methodology estimates the long-run effect of the LMRI on youth unemployment, I have deleted the dummy variables from our analysis.
